# Characterization of the ZFX family of transcription factors that bind downstream of the start site of CpG island promoters

**DOI:** 10.1093/nar/gkaa384

**Published:** 2020-05-14

**Authors:** Weiya Ni, Andrew A Perez, Shannon Schreiner, Charles M Nicolet, Peggy J Farnham

**Affiliations:** Department of Biochemistry and Molecular Medicine and the Norris Comprehensive Cancer Center, Keck School of Medicine, University of Southern California, Los Angeles, CA 90089, USA

## Abstract

Our study focuses on a family of ubiquitously expressed human C_2_H_2_ zinc finger proteins comprised of ZFX, ZFY and ZNF711. Although their protein structure suggests that ZFX, ZFY and ZNF711 are transcriptional regulators, the mechanisms by which they influence transcription have not yet been elucidated. We used CRISPR-mediated deletion to create bi-allelic knockouts of ZFX and/or ZNF711 in female HEK293T cells (which naturally lack ZFY). We found that loss of either ZFX or ZNF711 reduced cell growth and that the double knockout cells have major defects in proliferation. RNA-seq analysis revealed that thousands of genes showed altered expression in the double knockout clones, suggesting that these TFs are critical regulators of the transcriptome. To gain insight into how these TFs regulate transcription, we created mutant ZFX proteins and analyzed them for DNA binding and transactivation capability. We found that zinc fingers 11–13 are necessary and sufficient for DNA binding and, in combination with the N terminal region, constitute a functional transactivator. Our functional analyses of the ZFX family provides important new insights into transcriptional regulation in human cells by members of the large, but under-studied family of C_2_H_2_ zinc finger proteins.

## INTRODUCTION

RNA Polymerase 2 (Pol2)-mediated gene regulation is achieved, in part, by transcription factors (TFs) binding to a core promoter, defined as a region ±50 bp from the transcription start site (TSS) of a gene (1–4). Core promoters are composed of common sequence elements such as a TATA box or a CpG island (which is a genomic region with high GC content and a high density of CpG dinucleotides). TATA box-containing promoters often produce cell type-specific or induced (e.g. by a hormone) transcripts, whereas housekeeping genes are often driven by CpG island promoters (5). Both types of core promoters are bound by general TFs such as Pol2 and other components of the pre-initiation complex. However, a core promoter alone does not provide robust transcription, due to unstable interactions of the general transcriptional machinery with the DNA. Promoter activity can be increased by the action of site-specific, DNA-binding TFs that either bind proximal to the core promoter, stabilizing the recruitment of the transcriptional machinery, or to distal enhancer elements, bringing specific co-regulators to the core promoter via long-range chromatin looping (6).

There are ∼1600 TFs that have sequence-specific DNA binding properties (7,8). Alterations in gene expression caused by the inappropriate level, structure, or function of a site-specific, DNA-binding TF have been associated with a diverse set of human diseases, including cancers and developmental disorders (7,9,10), indicating the importance of understanding the normal and abnormal functions of these regulatory proteins. Site-specific DNA-binding TFs are classified according to their DNA binding domains, which provide useful information concerning their DNA binding patterns and their evolutionary relatedness (7). C_2_H_2_ zinc fingers (ZFs) comprise the largest class of site-specific DNA binding proteins encoded in the human genome (11); of the ∼1600 predicted human DNA binding transcription factors, 747 contain C_2_H_2_ zinc finger domains (8). This abundance suggests that the C_2_H_2_ zinc finger proteins (ZNFs) may be critical regulators of a large number of important biological networks. However, the majority of these TFs have not been well-studied, due to issues related to low expression levels, poor antibody quality, and a lack of knowledge as to what tissue or physiological processes they may regulate.

**Figure 1.**
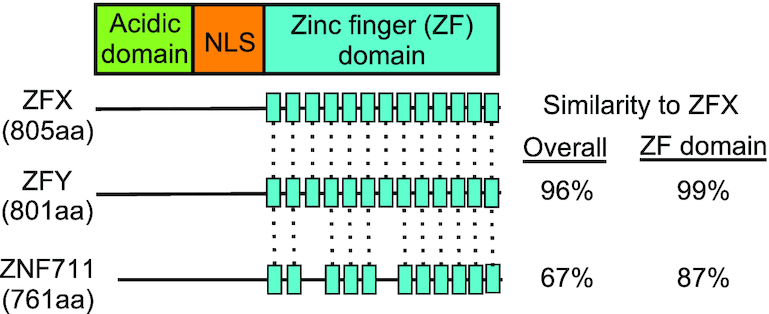
The ZFX gene family. Shown are gene structure schematics for ZFX, ZFY and ZNF711. Dashed lines indicate zinc fingers conserved between ZFX and the other two family members. NLS: nuclear localization sequence.

Our studies have focused on a small family of human C_2_H_2_ ZNFs that are ubiquitously expressed in human tissues. A Treefam (http://www.treefam.org) analysis reveals that members of the family include ZFX, ZFY and ZNF711 (Supplementary Figure S1A). ZFX and ZFY are nearly identical proteins encoded on either the X or Y chromosome, respectively (having 96% overall similarity, with 99% similarity in the zinc finger domains). ZNF711 is highly related to the other two family members, having 67% overall similarity with ZFX and 87% similarity in the zinc finger domains (Figure 1). Although previous studies have recognized the high similarity of ZFX and ZFY (12), the relationship of ZNF711 to ZFX and ZFY has only been recently noted (13). The next closest human ZNF identified by the Treefam analysis is ZNF639. However, we have not included ZNF639 in the ZFX family because it has only a 25% similarity to ZFX. ZFX and ZFY have 13 zinc finger domains at the C-terminal end of the protein; ZNF711 has amino acid differences that disrupt ZF3 and ZF7 and thus has only 11 ZFs. All 3 proteins have an acidic domain at the N-terminus and a nuclear localization signal between the acidic domain and the zinc finger domains; see Supplementary Figure S1B for a comparison of the amino acid sequences of the ZFX family members.

Of the three family members, ZFX has been the most studied in relation to a variety of human cancers. In fact, it has been implicated in the initiation or progression of many different types of human cancers, including prostate cancer, breast cancer, colorectal cancer, glioma, renal carcinoma, gastric cancer, gallbladder adenocarcinoma, non-small cell lung carcinoma and laryngeal squamous cell carcinoma (14–23). In these previous studies, it was shown that high expression of ZFX correlates with poor survival of cancer patients. Based on its increased levels and association with poor survival in many different cancer types, ZFX does not appear to be a tumor type-specific oncogene, but rather increased levels of ZFX (and perhaps also ZFY and ZNF711) may generally contribute to metaplastic transformation via causing tumor-promoting changes in the transcriptome. However, the mechanism(s) by which the ZFX family influences transcriptional regulation has not been determined. Therefore, we created knockout cells lacking expression of all ZFX family members, identified genes responsive to loss of these TFs, characterized and compared the binding patterns of ZFX, ZFY, and ZNF711 using ChIP-seq and ChIP-exo, and performed structure–functional analyses of the ZFX protein, identifying regions sufficient for DNA binding and transactivation.

## MATERIALS AND METHODS

### Overall design study

We created single and double knockout clones lacking ZFX and ZNF711 from female HEK293T cells (which naturally lack ZFY) and performed RNA-seq to examine effects on the transcriptome. We also performed ChIP-seq (extending our studies to include a male cell line to allow analysis of all three family members) and ChIP-exo to identify direct target genes of these TFs. We classified the ZFX family member binding sites using all known TSS from GENCODE release 19 (GRCH37.p19) and known CpG islands from UCSC table browser (http://genome.ucsc.edu/cgi-bin/hgTables). Finally, we created a series of FLAG-tagged ZFX mutant proteins and assayed the mutant proteins for DNA binding and transcriptional activity. A list of all genomic datasets used in this study can be found in Supplementary Table S1.

### Cell culture

Human kidney HEK293T (ATCC #CRL-3216) and prostate cancer 22Rv-1 (ATCC #CCL-2505) cells were obtained from ATCC (https://www.atcc.org/). Cells were cultured in appropriate media (HEK293T in DMEM and 22Rv1 in RPMI 1640) supplemented with 10% fetal bovine serum (Gibco by Thermo Fisher #10437036) plus 1% penicillin and 1% streptomycin at 37°C with 5% CO_2_. Cell lines were authenticated via the STR method and validated to be mycoplasma free using a universal mycoplasma detection kit (ATCC #30-1012K).

### CRISPR/Cas9-mediated genomic deletions

**Figure 2.**
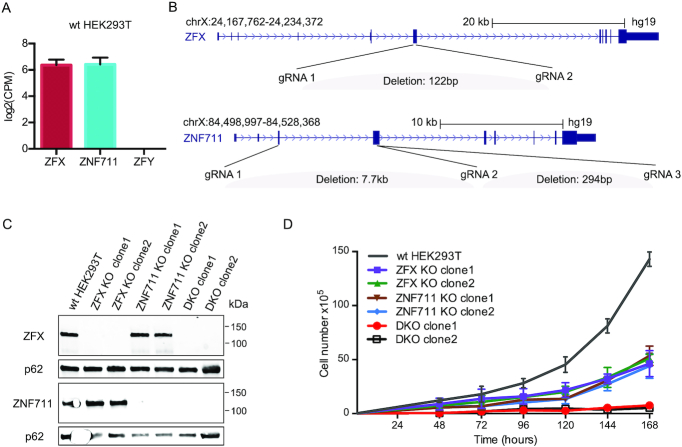
Loss of ZFX and ZNF711 in HEK293T cells inhibits cell proliferation. (**A**) Expression levels of ZFX/ZFY/ZNF711 in wt HEK293T cells. (**B**) Locations of gRNAs used to create CRISPR/Cas9-mediated ZFX and/or ZNF711 knockouts. The deletion of ZFX in ZFX KO clone1 and clone2 and the DKO clones were generated using ZFX gRNA1 and gRNA2. The deletion of ZNF711 in ZNF711 KO clone1 and the DKO clones was generated using ZNF711 gRNA1 and gRNA2; the deletion of ZNF711 KO clone2 was generated using ZNF711 gRNA2 and gRNA3. (**C**) Western blots showing the protein levels of ZFX and ZNF711 in wt HEK293T, ZFX KO clones, ZNF711 KO clones, and DKO clones; also shown is the level of p62 as a loading control. (**D**) Proliferation assays using wt HEK293T, two different ZFX and two different ZNF711 KO clones, and two DKO clones; data points are the mean of three biological replicates.

Guide RNAs used to create ZFX and ZNF711 functional deletions (see Supplementary Table S2) were cloned into pSpCas9(BB)-2A-Puro (PX459) V2.0 plasmid (Addgene #62988). HEK293T cells were transfected with PX459 V2.0 expressing Cas9 plus the gRNAs or with the PX459 V2.0 vector only (which expressed Cas9 but not guide RNAs) using Lipofectamine 3000 (Thermo Fisher #L3000015), according to the manufacturer's protocol. Twenty four hours after transfection, cells were selected with 2 ng/ul puromycin for 24 h and then harvested. Post-selection cell pools are stained with DAPI (Thermo Fisher #62248) and sorted for live cells using BD FACSAria Ilu SORP (USC Flow Cytometry Facility). Live single cells were sorted individually into a well of 96-well plates containing growth media for HEK293T (described above). Genomic DNA of single cell-derived clonal populations was extracted using QuickExtract DNA Extraction Solution (Epicentre #QE9050), following the manufacturer's protocol and was used in PCR-based homozygous deletion screening assays with primers listed in Supplementary Table S2. We identified multiple colonies that showed complete deletion of the DNA between the paired guide RNAs (not shown). RNA from those single cell-derived clonal populations was harvested using DirectZol RNA MiniPrep kit (Zymo #R2052) according to the manufacturer's protocol. cDNA was synthesized using the SuperScript VILO cDNA Synthesis Kit (Life Technologies #11754-050) following the manufacturer's protocol and used in qPCR-based (Quantabio #95054-02K) assays with primers listed in Supplementary Table S2. These assays demonstrated that there was no detectable RNA corresponding to the region within the deleted coding regions (not shown). Finally, a western blot was performed to demonstrate that there was no expression of ZFX or ZNF711 protein in the clones (see Figure 2C).

### Cell cycle analysis

Cells of wt HEK293T, two ZFX knockout (KO) clones, two ZNF711 KO clones, and three ZFX and ZNF711 double knockout (DKO) clones were treated with 70% ethanol for 2 h on ice, washed twice with cold PBS, and then labeled with DAPI (Thermo Fisher #62248) at a final concentration of 10 ug/ml for 30 min on ice, protected from light. The flow cytometry assay was performed using BD LSR II (USC Flow Cytometry Facility). Fixed cells were gated on single cells via Width and Area signals. Cell cycle analysis of the percentage of G0/G1, S and G2/M phases were calculated from the DAPI-area histogram using ImageJ (https://imagej.nih.gov/ij/).

### RNA-seq

Total RNA was extracted using DirectZol RNA MiniPrep kit (Zymo #R2052) following the manufacturer's protocol. RNA integrity was checked using RNA 6000 Nano kit (Agilent Technologies #50671511) on a 2100 Bioanalyzer (Agilent Technologies #G2939AA). RNA-seq libraries for controls, ZFX and ZNF711 KO clones, and the DKO clones were made using the KAPA Stranded mRNA kit with beads (Roche #KK8421) following the manufacturer's protocol. Samples were sequenced on an Illumina HiSeq3000 with 50 bp single-ended reads. The RNA-seq libraries of DKO cells transfected with a control plasmid, wt ZFX FLAG, or ZFX ZF11-13 FLAG were prepared by Novogene. Paired-end sequencing was performed by the company. RNA-seq results were aligned to GENCODE v19 and reads were counted using STAR (https://github.com/alexdobin/STAR). Differentially expressed genes with absolute fold change >1.5 were determined using edgeR (https://bioconductor.org/packages/release/bioc/html/edgeR.html). DAVID (https://david.ncifcrf.gov/summary.jsp) was used for gene ontology analyses; specifically, the Functional Annotation Clustering tool and the INTERPRO protein domain category was used, with default settings (three genes required per category) and medium stringency.

### Construction of ZFX zinc finger deletion mutants

ZFX mutant expression constructs were generated by amplifying the ZFX-Myc-DDK expression vector (Origene #RC214045) using primers with 15 bp complementary overhangs flanking different ZFs to create constructs containing ZF1-8, ZF9-13, ZF9-11, ZF11-13 or no ZF (see Supplementary Table S2). The resulting constructs were transformed into CopyCutter™ EPI400™ Chemically Competent E. coli (Lucigen #C400CH10) and induced to high copy number according to the manufacturer's protocol. Plasmids were purified using Qiagen miniprep kit (Qiagen #D4068) and the deletions were validated via Sanger sequencing. Primers used for cloning and sequencing are listed in Supplementary Table S2.

### Transient transfection assays

To test transcriptional activity of the ZFX deletion mutants, HEK293T cells were seeded into six-well plates and transfected during log phase growth. Transfection was carried out with Lipofectamine 3000 (ThermoFisher #L3000015) according to manufacturer's instructions. After 24 h, cells were lysed in TRI Reagent (Zymo #R2050-1-200) and RNA was recovered by precipitation. Total RNA was converted to cDNA using iScript (Bio-Rad #1708841BUN). RT-qPCR was carried out using SYBR on a BioRad CFX 1000. Data points represent results from triplicate wells and duplicate RT-qPCR readings. Primers used to monitor expression of endogenous genes are provided in Supplementary Table S2.

### Chromatin Immunoprecipitation Sequencing (ChIP-seq)

ZFX (Cell Signaling Technology # 5419S), ZNF711 (24) and ZFY (Sigma #SAB2102775-100UL) antibodies were used for ChIP assays in HEK293T and 22Rv1 cells, as previously described (7). 400–900 ug chromatin was used for ZFX (30 ul antibody), ZNF711 (5 ug antibody), and ZFY (10 ul antibody) ChIP assays. For ZFX and ZNF711 antibody validation, western blots were performed in wildtype and knockout cells. For ZFY antibody validation, we demonstrated that ZFY can be ChIPed in male 22Rv1 cells but not in female HEK293T cells, thus demonstrating that there is no cross reactivity with the other two family members (Supplementary Figure S1C). All ChIP-seq samples for endogenous TFs were performed in duplicate, following ENCODE standards. ChIP-seq libraries were prepared using the KAPA HyperPrep kit (Roche #KK8503) following the manufacturer's protocol. Samples were sequenced on an Illumina HiSeq3000 machine using 100 bp paired-end reads for ZFX and 50 bp single-end reads for all other samples. All ChIP-seq data were processed according to the ENCODE3 ChIP-seq pipeline (https://www.encodeproject.org/chip-seq/), and mapped to hg19; all data passed ENCODE quality standards. ChIP-seq peaks were called using MACS2 (https://github.com/taoliu/MACS), followed by identifying common peaks between duplicates using IDR (https://github.com/nboley/idr). To test DNA binding activity of mutant ZFX proteins, HEK293T cells were transfected with a plasmid expressing a FLAG-tagged wt ZFX or a mutated ZFX construct using Lipofectamine 3000 (Thermo Fisher #L3000015) according to the manufacturer's protocol. Cells were harvested 24 h after transfection for ChIP assays. For each ChIP assay, 5 ug of FLAG antibody (Sigma-Aldrich #F1804-200UG) was used with 150 ug chromatin. Also, 40 ug of chromatin, along with an antibody to H3K36me3 (Cell Signaling Technology #9763S), was used for ChIP-seq analysis of wt HEK293T and three DKO clones; the antibody was validated by the company to demonstrate no cross-reactivity to unmodified, mono- or di-methylated H3K36. ChIP-seq was performed and analyzed as described above.

### ChIP-exo

Approximately 100 million HEK293T cells were crosslinked for each ChIP-exo assay using the ChIP-seq protocol described above. Crosslinked cells, ZFX antibody (Cell Signaling Technology # 5419S), and ZNF711 antibody (Thermo Fisher #PA5-31815) were sent to Peconic, where the ChIP-exo assay was performed (http://www.peconicgenomics.com/services.html). Samples were sequenced on an Illumina NextSeq 500 machine using 2 × 40 bp paired-end sequencing generating ∼40 million reads per sample. Sequence reads were aligned to human (hg19) genome using using bwa-mem (v0.7.9a) (http://bio-bwa.sourceforge.net/). Peaks in ChIP-exo data were called using ChExMix (http://mahonylab.org/software/chexmix/).

### DNA methylation EPIC arrays

500 ng genomic DNA was extracted from wt HEK293T cells and the three DKO clones using the Zymo Quick-DNA Miniprep kit (Zymo #D3024) and bisulfite-converted using the Zymo EZ DNA Methylation kit (Zymo #D5001) according to the manufacturer's protocol. The bisulfite-converted DNA was analyzed using Illumina EPIC BeadArrays, as described (46). The BeadArrays were scanned and the raw signal intensities were extracted from the *.IDAT files using the ‘noob’ function in the minfi R package. The beta value (a measure of change in DNA methylation) was calculated as (M/(M+U)), in which M and U refer to the (pre-processed) mean methylated and unmethylated probe signal intensities, respectively. Measurements in which the fluorescent intensity was not statistically significantly above background signal (detection *P* value > 0.05) were removed from the dataset. Probes located from –1500 bp relative to the TSS and extending through the first coding exon (using the Illumina MethylationEPIC Manifest RefGene annotation) were included in the analysis as a defined set of ‘promoter’ probes for downstream analysis. The cut off used for identifying hypomethylated or hypermethylated probes was 0.2 for the absolute beta value difference between the methylation level of a probe in the DKO cells versus the wt HEK293T cells.

## RESULTS

### Loss of ZFX and ZNF711 inhibits cell proliferation and causes large changes in the transcriptome of HEK293T cells

For our initial investigations into the function of the ZFX family, we used the CRISPR/Cas9 system to functionally inactivate the ZFX and ZNF711 genes in female HEK293T cells. We chose to use these cells because they express similar levels of ZFX and ZNF711 (Figure 2A) but lack ZFY (which is encoded on the Y chromosome). Because ZFX and ZFY are so similar (96% overall), it is likely they have a similar function and the use of female cells meant that we only had to delete two TFs and not three to study the consequences of loss of the entire family. Paired sets of plasmids encoding guide RNAs designed to delete specific coding regions of ZFX or ZNF711 (Figure 2A) and co-expressing Cas9 were transfected into HEK293T cells; after 48 h individual cells were isolated using flow cytometry and then grown into colonies. Genomic DNA was extracted and analyzed using specific primers that spanned the deletion region (see Supplementary Table S2 for the sequence of all guide RNAs and primers used in this study). We identified multiple colonies that showed no expression of ZFX or ZNF711 (Figure 2C). However, our initial transfections did not produce any cells lacking both ZFX and ZNF711, despite screening a large number of colonies. Therefore, we next transfected guide RNAs that target ZFX into the ZNF711 knockout (KO) clone1 and selected single cell-derived colonies, this time using conditioned media (70% regular growth media plus 30% filtered used growth media) to provide a more supportive growth environment. We obtained several double knockout (DKO) cell clones that lacked expression of both ZFX and ZNF711 (Figure 2C). The difficulty in obtaining DKO clones suggested that reduction of both ZFX and ZNF711 may have negatively affected cell proliferation. To test this hypothesis, we performed proliferation assays over a 168-hr time course. As shown in Figure 2D, loss of either ZFX or ZNF711 reduced the proliferation rate of HEK293T cells to approximately the same level, whereas loss of both ZFX and ZNF711 caused a severe inhibition of cell proliferation; in general, we have observed that DKO cells grow slowly and must be kept at a high density to maintain viable cell populations.

**Figure 3.**
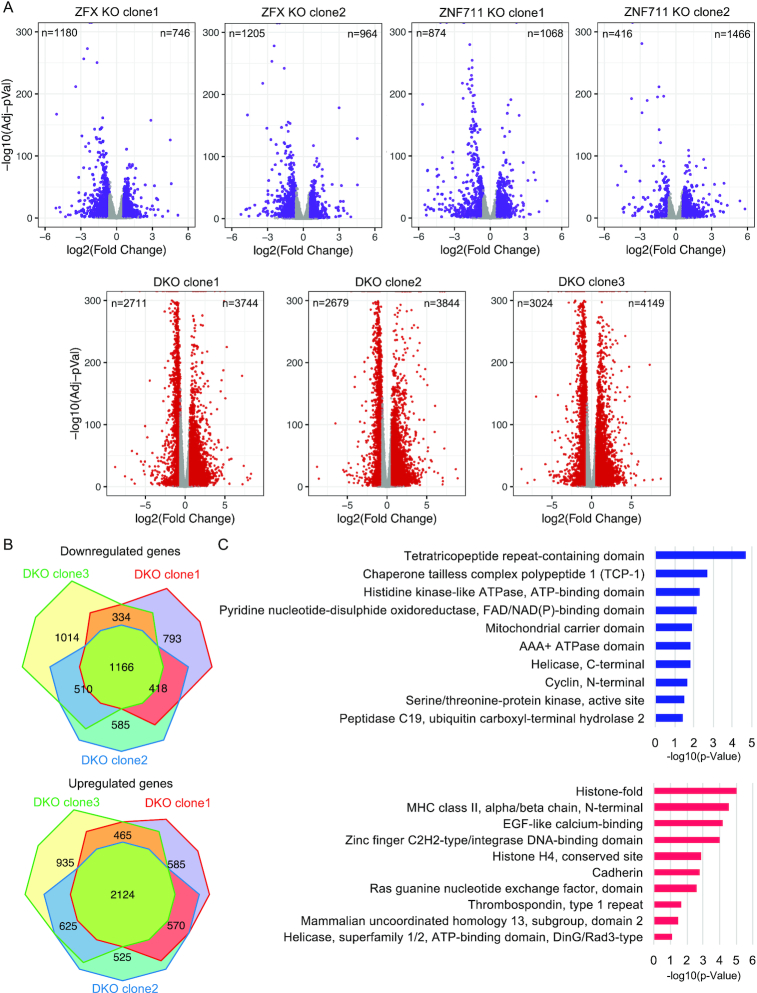
Reduction in ZFX and ZNF711 levels causes large effects on the transcriptome. (**A**) Volcano plots showing the differentially expressed genes (DEGs) identified via RNA-seq in comparisons of wt HEK293T versus ZFX KO clone1, KO clone2, ZNF711 KO clone1, KO clone2, DKO clone1, DKO clone2, or DKO clone3. (**B**) Comparison of DEGs commonly downregulated or upregulated in all three DKO clones. (**C**) Gene ontology analysis of the 1166 commonly downregulated and 2124 commonly upregulated genes in all three DKO clones.

The severe effects on proliferation in the ZFX and ZNF711 KO and DKO cells suggested that loss of these TFs was likely to cause major changes in the transcriptome of HEK293T cells. To test this hypothesis, we performed RNA-seq analysis of two ZFX KO clones, two ZNF711 KO clones, three DKO clones lacking both ZFX and ZNF711, and controls; each clone was analyzed using 3 biological replicates (producing 24 RNA-seq datasets in total). Volcano plots showing the differentially expressed genes (DEGs) in both of the ZFX KO clones, both of the ZNF711 KO clones, and the three DKO clones are shown in Figure 3A; see Supplementary Table S3 for the gene expression changes in all single and double knockout clones. In general, we observed that cells lacking ZNF711 but retaining ZFX had fewer changes in the transcriptome than did cells lacking ZFX but retaining ZNF711; cells lacking both TFs showed the greatest number of upregulated and downregulated genes. To address any potential issues due to clonal variation, we compared the genes showing altered regulation in each of the 3 individually derived clonal populations that lacked both ZFX and ZNF711, identifying 2428 genes downregulated in at least two of the 3 DKO clones and 1166 genes commonly downregulated in all three DKO clones (Figure 3B). We also identified 3784 genes upregulated in at least two of the three DKO clones and 2124 genes commonly upregulated in all three of the DKO clones. Gene ontology analyses of the commonly deregulated genes in all three DKO clones revealed that different categories of genes were upregulated versus downregulated (Figure 3C). For example, genes that are upregulated upon loss of ZFX and ZNF711 include histone genes, zinc finger TFs and cadherins whereas genes that are downregulated upon loss of the two TFs include kinases, ATPase, peptidases, chaperone proteins, and oxidoreductases. A complete list of the clusters and all genes identified in each cluster can be found in Supplementary Table S3J and K. In support of our finding that loss of ZFX and ZNF711 resulted in proliferation defects, the term ‘Cyclins and Cell Cycle Regulation’ was one of the top identified pathways in the set of downregulated genes; additionally, flow cytometry cell cycle analysis revealed that the DKO cells have a higher percentage of G0/G1 cells and a lower percentage of G2/M cells than wt HEK293T cells (see Supplementary Figure S2).

### ZFX family members have essentially identical binding patterns at CpG island promoters

**Figure 4.**
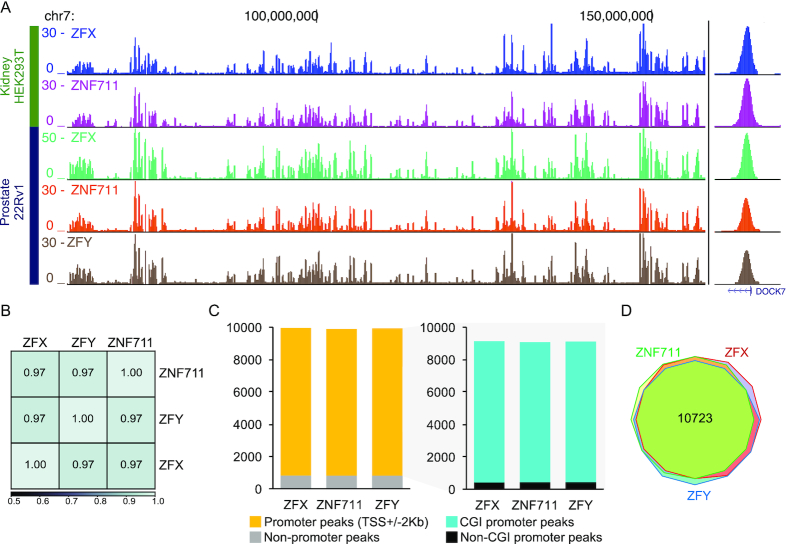
ZFX family members have essentially identical binding patterns at CpG island promoters. (**A**) Browser tracks showing ZFX family member binding profiles in female HEK293T kidney cells and male 22Rv1 prostate cells. Also shown is a zoom in on a single peak located in the DOCK7 promoter region. (**B**) Shown is a heatmap illustrating the genome-wide correlation of ZFX family member binding patterns in 22Rv1 cells. (**C**) Bar graph of genomic distributions of ZFX family member binding sites in 22Rv1 cells in promoter and non-promoter regions (left) and bar graph showing the relative distribution of binding sites in CpG island (CGI) promoters and non-CpG island promoters (right). (**D**) Venn diagram comparing the sets of CpG island promoters bound by ZFX, ZFY and ZNF711 in 22Rv1 cells.

Our next step in characterizing ZFX and ZNF711 was to define their genome-wide binding profiles by performing ChIP-seq in HEK293T cells using antibodies to ZFX and ZNF711; we note that the antibodies we used for these experiments have passed ENCODE validation criteria, as all signal on a Western blot is eliminated in the individual knockout clones (Figure 2C). All ChIP-seq experiments were performed using biological duplicates (see Supplementary Table S1); browser tracks from a single replicate of ZFX and ZNF711 ChIP-seq are shown in Figure 4A. We found that the binding profiles are very similar for ZFX and ZNF711. As noted in Figure 1, ZFY is also highly related to ZFX and, based on the binding profiles of ZFX and ZNF711, one might expect that ZFY would also have a similar binding pattern as ZFX. However, ZFY is not expressed in female HEK293T cells. To allow a comparison of the binding patterns of ZFX, ZFY, and ZNF711, we next performed replicate ChIP-seq experiments in male 22Rv1 prostate cells for all three family members (ZFY antibody validation was performed by demonstrating that no signal was detected by ChIP using female HEK293T cells). We found that all three family members showed highly correlated binding patterns throughout the human genome (Figure 4A, B). Peaks were identified for all ChIP-seq datasets and annotated into promoter vs. non-promoter binding sites. We found that each factor binds mainly to promoters that are CpG islands (Figure 4C). The CpG island promoters bound by the three factors are essentially the same, with a total of 10 723 CpG island promoters bound by the union of ZFX, ZFY and ZNF711(Figure 4D), corresponding to 72% of the active CpG island promoters in 22Rv1 cells.

### ZFX and ZNF711 have properties of a transcription activator when bound downstream of the TSS

**Figure 5.**
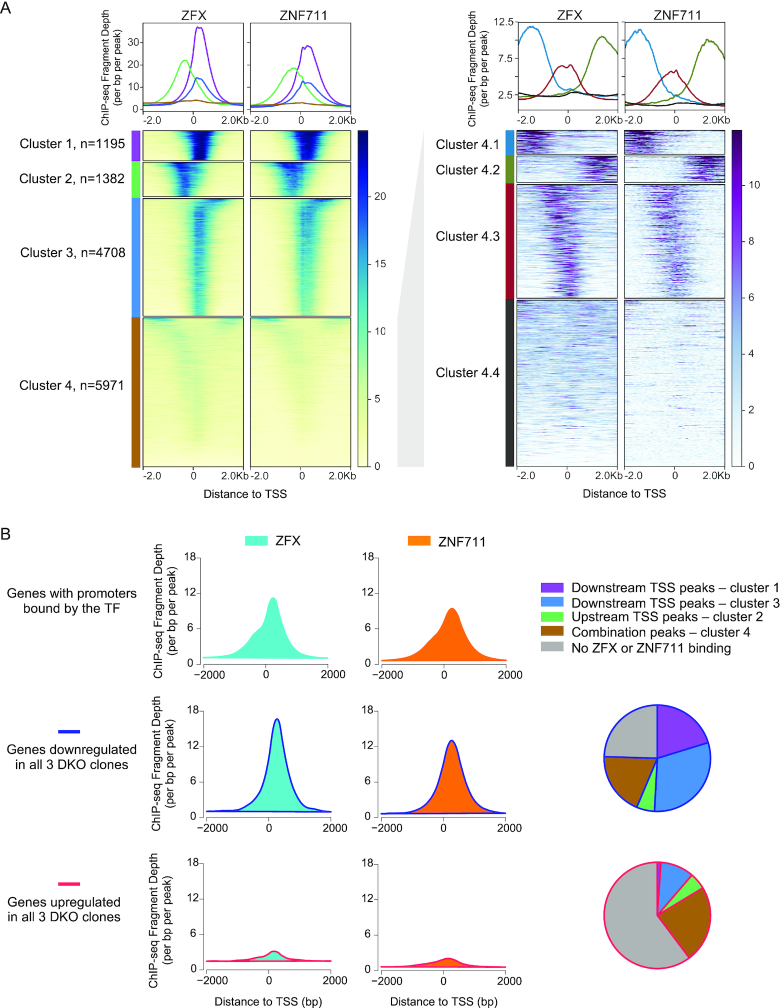
ZFX and ZNF711 have properties of a transcription activator when bound downstream of the TSS. (**A**) ZFX and ZNF711 peak sets from HEK293T cells were clustered using K-means clustering, identifying four sets of peaks with distinct binding sites (left); cluster 4 (combination peaks) was subsequently re-clustered, identifying 4 subsets (right). Tag density plots for each of the 4 different clusters are presented on top of the heatmaps. (**B**) Average signals of ZFX and ZNF711 ChIP-seq reads in wt HEK293T at all promoters bound by each TF (top), promoters of genes with decreased expression in all three DKO clones (middle), and promoters of genes with increased expressions in all three DKO clones (bottom). Also shown, for both the downregulated and the upregulated gene categories, is the percentage of genes whose promoters are bound by ZFX or ZNF711 in peak categories 1–4, or not bound by ZFX or ZNF711.

The binding patterns shown above demonstrate that ZFX family members bind to CpG island promoters. To further investigate the binding pattern of these TFs, we performed a *K*-means clustering based on the peak locations relative to the nearest TSS, identifying four groups of binding sites for ZFX and ZNF711 (Figure 5A). Interestingly, the strongest binding sites comprise ∼1200 peaks (cluster 1) which are located downstream of the TSS. An additional larger set of ∼4700 peaks (cluster 3) has a similar downstream location, but a slightly weaker binding profile. We also identified ∼1400 peaks (cluster 2) that are located upstream of the TSS and a set of weaker peaks (cluster 4) that appear to have a Y-shaped pattern. Further analysis of the peaks in cluster 4 revealed peaks that are upstream (cluster 4.1), downstream (cluster 4.2), and over the TSS (cluster 4.3), as well as a set of peaks that are very small and have no distinct binding pattern (cluster 4.4). We note that the upstream and downstream peaks in cluster 4 have a different location than the peaks in clusters 1, 2 and 3. The peaks in clusters 1 and 3 are located downstream, but quite near, the TSS whereas the peaks in cluster 4.2 are much farther downstream (close to +2 kb). Similarly, the peaks in cluster 2 are located upstream, but near, the TSS whereas the peaks in cluster 4.1 are much farther upstream (close to –2 kb).

The fact that most of the strongest ZFX and ZNF711 peaks are downstream of the TSS (clusters 1 and 3) raises several questions. For example, do these TFs regulate transcription from a location downstream of the TSS or is regulation achieved only when the TFs are bound to the minority of sites upstream of the TSS? Also, do the TFs function as direct activators or repressors and, if so, does their activity differ depending on the binding location? To answer these questions, we compared the binding profiles of ZFX and ZNF711 at all bound promoters and at promoters that we identified as commonly downregulated or upregulated in all 3 DKO clones (Figure 5B). The tag density plots of all ZFX or ZNF711 peaks were quite broad and showed a large peak at +240 and a shoulder at –240. Interestingly, the promoters that are downregulated upon loss of ZFX and ZNF711 have very strong peaks downstream of the TSS with a frequency peak at +240, suggesting that ZFX and ZNF711 function as activators when bound downstream of the TSS on that group of promoters. In contrast, promoters that are upregulated upon loss of ZFX and ZNF711 have very flat binding profiles, suggesting that genes that show increased expression in the DKO cells are indirectly regulated by ZFX and ZNF711, perhaps because they are components of affected signaling pathways. The pie charts show the percentage of deregulated genes that have promoters bound by ZFX or ZNF711, broken into the different clusters; in total, 86% of the downregulated genes are bound by ZFX or ZNF711 whereas only 24% of the upregulated genes are bound by ZFX or ZNF711 (and most of these have peaks located in the weaker cluster 4). Therefore, ZFX and ZNF711 appear to function mainly as transcriptional activators, but only when they are bound downstream of the TSS.

### ZFX family members bind throughout the first several hundred base pairs of the transcribed region of their target genes

**Figure 6.**
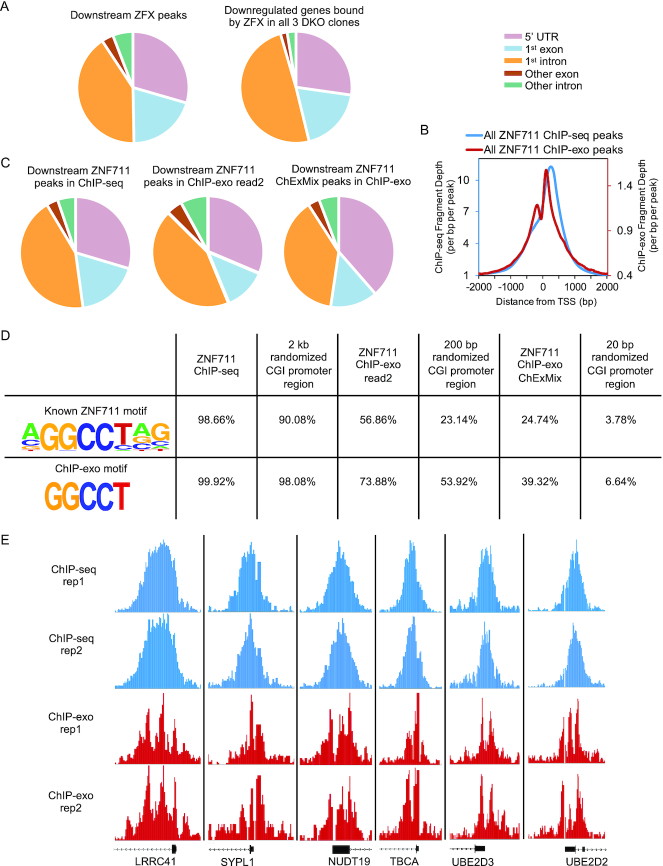
Characterization of ZFX and ZNF711 binding sites. (**A**) Classification of binding sites based on genomic locations of all ZFX peaks located downstream of the TSS and ZFX downstream peaks at promoters of genes down-regulated in all 3 DKO clones. (**B**) Classification of ZNF711 downstream ChIP-seq peaks, downstream peaks identified using read2 of the ChIP-exo dataset, and downstream peaks identified by the ChexMix program (+/-10 nt from the nt identified as the binding site by the program). (**C**) Tag density plots of all ZNF711 peaks from standard ChIP-seq and from ChIP-exo. (**D**) Motif analysis using the top 5000 peaks identified from standard ZNF711 ChIP-seq (average width 1800 nt), ChIP-exo read2 (average width 300 nt), ChIP-exo by the ChexMix program (20 nt), and randomized CpG island promoter regions (width 2 kb, 200 bp, and 20 nt). The peaks were searched for the known ZNF711 motif and the 5 nt motif identified by ChIP-exo. (**E)** Zoom-in comparison of peaks from ZNF711 standard ChIP-seq replicates and ChIP-exo replicates.

Because the majority of the ZFX binding sites occur downstream of the TSS within the transcribed region, we annotated the position of the downstream ZFX binding sites relative to gene structure (Figure 6A). We found that most of these binding sites fall within the 5′ UTR, the first coding exon, or the first intron, suggesting that there was not a preference for binding to coding or non-coding regions downstream of the TSS. This was true for the set of all ZFX peaks and for the set of ZFX peaks found at the genes that are commonly downregulated in all three of the DKO clones. However, although we used the genomic location of the summit of the called ChIP-seq peaks for the location analysis, the ‘genomic summit’ of a ChIP-seq peak does not necessarily correspond to the location of the precise binding site (e.g. due to the random nature of the sonication of the chromatin). The precise identification of a peak summit may also be compounded when analyzing ZFX and ZNF711. We note that the tag density plots shown in Figure 5 show a fairly broad binding profile for ZFX family members. Also, close inspection of single peaks reveals a relatively wide peak at individual promoters (see Figure 4A for the single ChIP-seq peak in the DOCK7 promoter). For comparison to another multi-finger ZNF, we calculated the average peak width of ZFX (13 ZFs) and CTCF (11 ZFs) peaks and found that the ZFX peaks (average width of 1816 bp) are quite a bit wider than the CTCF peaks (average width of 747 bp); the ChIP-seq experiments for both TFs were performed in our lab using the same protocol. The broad ZFX and ZNF711 peak widths suggested a need for a more precise delineation of the binding sites. Therefore, we used ChIP-exo, a modification of ChIP-seq that improves the resolution of binding sites (25). The use of ChIP-exo reduced the average width of the ZNF711 binding sites from ∼1800 to ∼300 bp, providing a more distinct pattern of upstream and downstream binding (Figure 6B). We compared the genomic locations of the wide ZNF711 ChIP-seq binding sites to the narrow ChIP-exo peaks (in both cases, using the peak summits obtained using the ENCODE pipeline). We also used peak information from ChExMix, a program designed specifically to identify precise binding sites from ChIP-exo data (Figure 6C). In all cases, the downstream ZNF711 binding sites are spread throughout the 5′UTR, first coding exon, and first intron. These results suggest that the localization of ZNF711 is not related to the classification of the transcribed region to which it binds.

Previous studies have identified a ZNF711 motif (AGGCCTAG) using ChIP-seq data from a brain tumor cell line SH-SY5Y (24). However, these studies used the entire ChIP-seq peak width (which, as shown above, covers a very large area of the proximal promoter region), making it difficult to be sure if the identified motif was involved in direct recruitment of ZNF711 or if it was instead a motif commonly found in CpG island promoters. Also, the ChExMix program, which is used to call motifs in ChIP-exo data, identified a smaller motif of GGCCT. This shorter motif is similar to a short motif GGCC identified for mouse Zfx using ChIP-seq data (26) and for ZFY using *in vitro* assays (27–29). To more precisely define the ZNF711 binding motif, we performed motif analysis using the top 5000 ZNF711 peaks identified by ChIP-seq (using the entire width of the MACS2 peaks), identified by ChIP-exo (using the entire width of the MACS2 peaks) or identified by the ChExMix program (in this case, because ChExMix outputs a single nt for each peak, the sequence was extended +/- 10bp for motif analysis). We found that essentially all of the top 5000 ZNF711 ChIP-seq peaks contain the known ZNF711 motif and the ChIP-exo GGCCT motif (Figure 6D). However, because the ZNF711 peaks are quite wide (∼2 kb), they span a large proportion of the promoter region. As shown in Figure 4, ZNF711 binds mainly to GC-rich CpG island promoters. This suggests that these motifs may have been identified because they are GC-rich and commonly found in CpG island promoters. In fact, when we analyzed 5000 2 kb randomized regions from CpG island promoters, we found that all 2 kb randomized promoter regions also contain these same motifs. As noted above, ChIP-exo reduced the peak widths to an average size of 200–300 nt. Motif analysis of the ChIP-exo peaks showed a reduction in the number of peaks that contained the known ZNF711 motif or the shorter GGCCT motif, although the peaks did have a higher percentage of both motifs than did randomly selected 200 bp regions from CpG island promoter downstream regions. Finally, analyzing the sequences +/- 10 nt from the ChExMix peak summits resulted in a further drop in the percentage of peaks that contain the motifs. In this case, ∼25% of the ChExMix peak locations contain the known ZNF711 peak and ∼40% contain the smaller GGCCT motif. However, of note, randomized 20b regions contain these motifs at a very low frequency (∼5%). These results suggest that the ZNF711 binding sites are enriched in both the known motif and the GGCCT motif, but the majority of sites do not contain either motif. We also note that both the ZNF711 motif and the GGCCT motif are present throughout the genome, albeit at a higher density in CpG islands (data not shown). Thus, the presence of a motif is perhaps supportive of binding but does not appear to be absolutely required nor sufficient for binding.

Visual inspection of individual promoters revealed that not only did the ChIP-exo method result in narrower peaks overall, but the broad ChIP-seq peaks were fractured into multiple peaks in the ChIP-exo datasets (Figure 6E). These results suggest that there are multiple ZNF711 binding events for each promoter. Due to limitations of the ChIP assay, we cannot distinguish between multiple ZNF711 molecules bound to a given promoter in the same cell or a single ZNF711 molecule binding at different locations in a given promoter in different cells. Perhaps the multiple copies of CCGGT elements within CpG island promoters simply help to localize ZFX family members to the region of open chromatin in a CpG island promoter, with the exact distance from the TSS not being important for regulation as long as binding is downstream of the TSS. We note that we performed similar ChIP-exo experiments using a ZFX antibody. Unfortunately, although the overall patterns were the same as for ZNF711, the ZFX antibody did not perform as well in ChIP-exo in either of two independent experiments (producing much smaller peaks overall, but in the same locations) and therefore this data was not included in our analyses.

As noted above, the ZFX family binds almost exclusively to CpG island promoters. Although the identified DNA binding motifs do not contain a methylatable CpG dinucleotide, there are many CpGs within each promoter region bound these TFs. Changes in the levels of DNA methylation can have major effects on promoter activity, with increased methylation leading to gene silencing (30,31). To address the question as to whether binding of ZFX and ZNF711 affects the DNA methylation level at target promoters, we performed DNA methylation assays using Illumina EPIC arrays for wt HEK293T cells and the three DKO cell lines. As shown in Supplementary Figure S3, we found that the loss of ZFX and ZNF711 results in a slight hypomethylation at many promoters, but that this overall promoter hypomethylation could not be specifically associated with ZFX- or ZNF711-mediated gene regulation.

### The first 10 C_2_H_2_ zinc fingers of ZFX are dispensable for DNA binding and transcriptional activity

**Figure 7.**
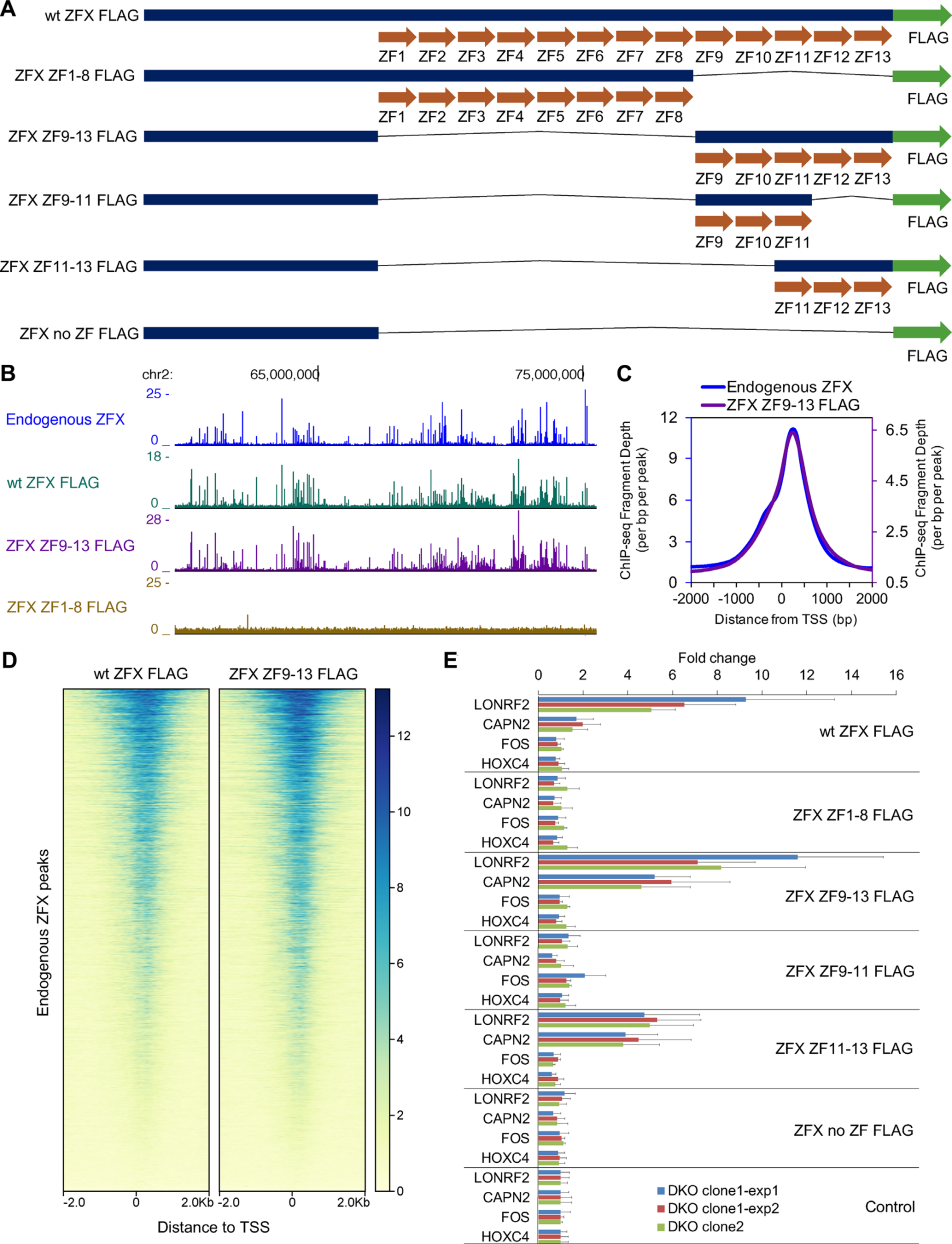
Functional analysis of the ZFX protein. (**A**) Schematic of FLAG-tagged ZFX zinc finger (ZF) mutant constructs. (**B**) Browser tracks showing genomic binding profiles of endogenous ZFX and FLAG-tagged wt ZFX and ZFX mutants in HEK293T cells. (**C**) Tag density plots of ChIP-seq peaks comparing endogenous ZFX and ZFX ZF9-13 peak locations in HEK293T cells. (**D**) Heatmaps showing ChIP-seq data from FLAG-tagged wt ZFX and ZFX ZF9-13 centered on the genomic locations of the endogenous ZFX peaks. (**E**) Expression levels following transfection with different ZFX constructs (as analyzed by RT-qPCR) of two genes (LONRF2 and CAPN2) whose promoters are bound by both ZFX and ZNF711 in wt HEK293T cells and which show a reduction in gene expression in all three DKO clones, of one gene (FOS) that is upregulated in all three DKO cells (a putative indirect target gene), and of one gene (HOXC4) that shows no expression changes in the DKO cells. Expression data were normalized to the control (cells transfected with an unrelated plasmid). Three independent experiments were performed using two different clonal populations of DKO cells; data points represent results from triplicate wells and duplicate PCR readings. Error bars indicate the pooled standard deviations of the means for the constructs and for the normalizing control.

As our next step, we wished to define which of the C_2_H_2_ ZFs were involved in recruitment of the ZFX family to chromatin. As noted above, ZFX and ZFY have 13 C_2_H_2_ ZFs but ZNF711 has amino acid changes that eliminate the C_2_H_2_ structure for ZF3 and ZF7 (Supplementary Figure S1), suggesting that perhaps ZFs closer to the C-terminus are used for DNA binding. To test this hypothesis, we created ZFX protein constructs that contained the N-terminus and only ZF1-8 or the N-terminus and only ZF9-13 (Figure 7A; Supplementary Figure S4A). Plasmids expressing FLAG-tagged versions of wt and mutant ZFX proteins were transfected into HEK293T and/or DKO cells, in vivo expression was confirmed by Western blot (Supplementary Figure S4B), and ChIP-seq was performed using a FLAG antibody. The FLAG-tagged wt ZFX produced a genomic binding pattern similar to the pattern obtained using the endogenous ZFX antibody, as did the FLAG-tagged ZFX that lacked ZF1-8 but contained ZF9-13 (Figure 7B). In contrast, FLAG-tagged ZFX containing ZF1-8 but lacking ZF9-13 did not bind to the genome, even though it was expressed at the same level as the FLAG-tagged wt ZFX. These results suggested that ZF9-13 are involved in binding. Many C_2_H_2_ ZNFs, such as the Sp1 and Kruppel-like family (KLF) members, use three ZFs to bind to DNA (4,32,33). Therefore, we next created additional mutant ZFX proteins, one containing only ZF9-11, one containing only ZF11-13, and one construct which lacked all ZF (no ZF). ChIP analysis revealed that ZF11-13 are sufficient for recruitment of ZFX to promoter regions (Supplementary Figure S4C). For comparison, we performed a prediction of the DNA binding motifs for the different ZFX mutant constructs using the website tool ‘DNA-binding Specificities of Cys_2_His_2_ Zinc Finger Proteins’ (http://zf.princeton.edu/); the predicted motif for ZFX ZF11-13 closely matches the motif identified using the ChIP-exo peaks (Supplementary Figure S5).

**Figure 8.**
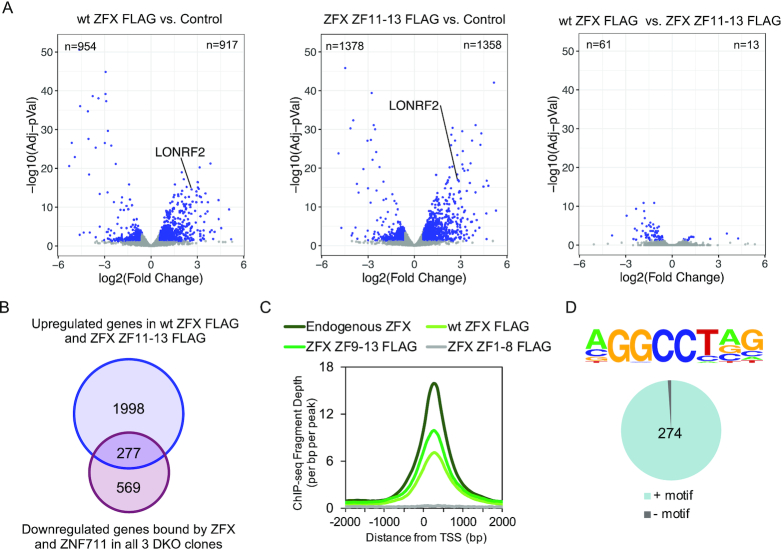
ZFX ZF11-13 has very similar transcriptional activities as wt ZFX. (**A**) Volcano plots showing the differentially expressed genes (DEGs) identified via RNA-seq in comparisons of DKO cells 24 hr after transfection with FLAG-tagged wt ZFX vs. a control plasmid, FLAG-tagged ZFX ZF11-13 vs. a control plasmid, and FLAG-tagged wt ZFX vs. FLAG-tagged ZFX ZF11-13. (**B**) Shown is a Venn diagram comparing the 846 genes that are bound by ZFX and ZNF711 in wt HEK293T cells and show a decrease in mRNA levels in all three DKO clones and the 2275 genes that show increased levels in DKO cells transfected with either FLAG-tagged wt ZFX or ZFX ZF11-13. (**C**) Shown is a tag density plot of ChIP-seq data for endogenous ZFX, FLAG-tagged wt ZFX, ZFX ZF9-13, or ZFX ZF1-8 at the set of 277 responding promoters identified in panel B. (**D**) Motif coverage analysis of the 277 responding promoters.

C_2_H_2_ ZFs have also been implicated in protein-protein interactions (34), suggesting that perhaps some of the ZFs not involved in genomic recruitment may be involved in transcriptional activity. To examine this possibility, we tested the ZFX constructs using a transient transfection reporter assay. ZFX expression constructs were transfected into DKO cells and the expression of endogenous genes was monitored by RT-qPCR after 24 h, using triplicate transfections for each data point. We examined expression of two genes (LONRF2 and CAPN2) whose promoters are bound by both ZFX and ZNF711 in wt HEK293T cells and which show a reduction in gene expression in all 3 DKO clones, of one gene (FOS) that is upregulated in the DKO cells (a putative indirect target gene), and of one gene (HOXC4) which shows no expression changes in the DKO cells. As shown in Figure 7C, we observed strong upregulation by a subset of the transfected ZFX constructs only for the two genes which are bound by ZFX in wt HEK293T cells and that show a reduction in RNA levels upon loss of ZFX family members (the putative direct target genes). This increased expression was observed in multiple, independent experiments using two independently derived DKO clones. The putative indirect target gene and the control gene were not affected upon transfection of the ZFX constructs. We observed that the ability of the ZFX constructs to bind to the genome was correlated with the ability to increase expression levels of the target genes. Because the FLAG-tagged ZFX ZF11-13 could increase expression of endogenous target genes as well as the FLAG-tagged wt ZFX construct, this suggests that the first 10 C_2_H_2_ ZFs are dispensable for genomic DNA binding and transcriptional activity (27). To further examine this possibility, we transfected FLAG-tagged wt ZFX, FLAG-tagged ZFX ZF11-13, or a control plasmid not expressing ZFX into DKO cells and compared global expression by RNA-seq (Figure 8). Volcano plots DEGs in DKO cells transfected with wt ZFX or ZFX ZF11-13, as compared to the control cells, are shown in Figure 8A; see Supplementary Table S3 for all DEGs. We identified thousands of genes that responded to the reintroduction of ZFX into the DKO cells. To further compare the cellular response to a 24 h exposure to wt ZFX versus ZFX ZF11-13, we created a volcano plot comparing these two datasets. We found that there are very few genes that show differential responses to the wt ZFX (containing 13 ZFs) versus the ZFX ZF11-13 (containing only the final three ZFs). To identify the putative direct target genes in DKO cells that are responsive to the reintroduction of ZFX, we compared the 846 genes that are bound by ZFX and ZNF711 in wt HEK293T cells and show a decrease in mRNA levels in all three DKO clones and the 2275 genes that show increased levels in DKO cells transfected with either FLAG-tagged wt ZFX or ZFX ZF11-13 (Figure 8B). We found 277 responding promoters. The binding patterns of transfected FLAG-tagged wt ZFX and FLAG-tagged ZFX ZF9-13 at the responding promoters (identified in Figure 8B) recapitulate the endogenous ZFX binding pattern, which has a peak at +240 downstream of the TSS (Figure 8C). We found that 274 of the 277 responding promoters have the known motif (Figure 8D).

## DISCUSSION

ZFX has been shown to have increased expression in tumors, with high expression negatively correlating with patient survival (14–23,35). The other 2 members of the ZFX family, ZFY and ZNF711, have not been as well-studied, especially in the cancer field. However, mutations in the ZNF711 coding sequence have been found to be associated with several families that display X-linked inherited mental retardation (24,36–38), suggesting that ZFX family members may be critical mediators of cell proliferation and/or have a role in tissue-specific differentiation. However, the previous studies of the ZFX family members have mainly been correlative analyses, providing essentially no insights into the mechanisms by which these TFs may function. We have used CRISPR-mediated deletion to demonstrate that loss of ZFX and ZNF711 (in cells naturally lacking ZFY) has severe defects in proliferation. Using a combination of ChIP-seq and RNA-seq, we have demonstrated that this 3-member family of C_2_H_2_ ZNFs activates transcription when bound downstream of the TSS in CpG island promoters. Because the ZFX family members bind to thousands of CpG island promoters, many of which regulate genes required for essential ‘housekeeping’ functions, it is possible that the these TFs act in a similar as manner as the MYC family of oncogenic transcription factors (39). Although MYC binds to thousands of promoters, it has been shown to have modest effects on the activity of any given promoter. Cells lacking MYC are impaired in proliferation; they can enter the cell cycle but soon cease to divide (40). Similarly, we show that cells lacking the ZFX family members have profound proliferation defects, but modest effects on the cell cycle parameters. We have shown that ZF11-13 are necessary and sufficient for DNA binding, and, in combination with the N terminal region, constitute a functional transactivator. We note that a previous study used an in vitro DNA binding assay to show that ZF12-13 of mouse Zfy2 can bind to a specific oligonucleotide containing an AGGCCY motif (27). However, it was not known if those ZFs of the ZFX family members would be capable of recruitment to, or stable interaction with, CpG island promoter regions. We have now shown that the last three ZFs of human ZFX have the capability of stable in vivo binding at target promoters in the context of a chromatin environment. Interestingly, ChIP-exo experiments suggest that the identified DNA binding motif may aid in recruiting ZFX family members to the genome but the presence of a motif under the direct binding site is neither sufficient nor, in some cases, necessary for genomic recruitment.

Although this study has extended our knowledge about this uncharacterized family of ZNF transcription factors, several crucial questions remain unanswered. (i) What distinguishes a responsive from a non-responsive target gene? For example, ∼10 000 CpG island promoters are bound by ZFX family members in a given cell type. However, less than half of the bound promoters show responsiveness to loss of ZFX and ZNF711 in the knockout HEK293T cells (Figure 3) or after knockdown of all three family members in 22Rv1 prostate cancer cells (data not shown). To date, we have not been able to identify differences in promoter structure, activity level, or epigenetic modifications that distinguish promoters that are bound by ZFX and ZNF711 and show decreased expression in the DKO cells from those that are bound and do not show decreased expression (data not shown). (ii) Is there functional significance that ∼75–80% of the binding sites in the promoters that are downregulated in the DKO cells are within the transcribed region? Certain ZNFs have been shown to be both RNA and DNA binding proteins (34,41–45). At first thought, this binding site distribution might suggest a role for ZFX in RNA processing. However, the binding sites are distributed throughout the 5′ UTR, the first coding exon, or the first intron. This distribution pattern makes it difficult to envision a role for ZFX in a post-transcriptional process such as splicing, unless ZFX can play a different role at different promoters. Perhaps a more reasonable possibility could be a role in RNA trafficking through interaction of ZFX with a site within the 5′ region of the transcripts. As an initial investigation into the possibility that ZFX regulates its target genes by binding to a GGCCU motif in the target RNAs, we performed eCLIP in FLAG-tagged wt ZFX transfected HEK293T cells using a FLAG antibody has been previously well-characterized to work in the eCLIP assay. However, we did not detect ZFX binding to the 5′ regions of RNAs encoded from the ZFX target promoters (data not shown). Another possibility is that, due to their binding to transcribed regions, ZFX and ZNF711 may be involved in transcriptional elongation. To test this possibility, we performed ChIP-seq for the elongation mark H3K36me3 in wt HEK293T cells and in the three DKO clones (Supplementary Figure S6). We found that promoters bound by ZFX and ZNF711 have much higher levels of H3K36me3 in wt HEK293T cells than do promoters not bound by these TFs. Interestingly, we found that the levels of H3K36me3 are reduced in the DKO cells at all promoters, not just at those bound by ZFX and ZNF711. Also, in the DKO cells, reduction of H3K36me3 occurs at genes that are downregulated and at genes that show no changes in expression. Therefore, it seems that ZFX family members may be important in recruiting an H3K36me3 histone methyltransferase to transcribed regions, but changes in the levels of this mark are not correlated with changes in gene expression. (iii) Are the ZFX C_2_H_2_ ZFs that are not required for genomic recruitment involved in other processes? ZFs have been implicated in protein-protein interactions (33). ZF1, ZF2, ZF4-6 and ZF8-10 are well-conserved between ZFX, ZFY and ZNF711 (Figure 1) and yet are not required for genomic recruitment. It is possible that interactions with co-regulators could be mediated through one or more of these non-DNA binding ZFs. However, transfection experiments suggest that ZFX ZF11-13 has very similar transcriptional activity as does wt ZFX, suggesting that co-activators may interact with the N-terminus of ZFX (which is conserved in the ZFX ZF11-13 construct). Future studies are required to completely understand how the ZFX family plays an essential role in normal and disease cellular physiology.

## DATA AVAILABILITY

The ChIP-seq, ChIP-exo, RNA-seq, and DNA methylation EPIC data are available in NCBI’s Gene Expression Omnibus (https://www.ncbi.nlm.nih.gov/geo) and are accessible through GEO Series accession number GSE145160.

## Supplementary Material

gkaa384_Supplemental_Files

## Notes

Present address: Andrew A. Perez, Division of Biology and Bioengineering, California Institute of Technology, Pasadena, CA 91125, USA.

## References

[1] Fuda N.J., Ardehali M.B., Lis J.T. Defining mechanisms that regulate RNA polymerase II transcription in vivo. Nature. 2009; 461:186–192. 19741698 10.1038/nature08449 PMC2833331

[2] Haberle V., Stark A. Eukaryotic core promoters and the functional basis of transcription initiation. Nat. Rev. Mol. Cell Biol. 2018; 19:621–637. 29946135 10.1038/s41580-018-0028-8 PMC6205604

[3] Core L., Adelman K. Promoter-proximal pausing of RNA polymerase II: a nexus of gene regulation. Genes Dev. 2019; 33:960–982. 31123063 10.1101/gad.325142.119 PMC6672056

[4] Vihervaara A., Duarte F.M., Lis J.T. Molecular mechanisms driving transcriptional stress responses. Nat. Rev. Genet. 2018; 19:385–397. 29556092 10.1038/s41576-018-0001-6 PMC6036639

[5] Saxonov S., Berg P., Brutlag D.L. A genome-wide analysis of CpG dinucleotides in the human genome distinguishes two distinct classes of promoters. Proc. Natl. Acad. Sci. U.S.A. 2006; 103:1412–1417. 16432200 10.1073/pnas.0510310103 PMC1345710

[6] Farnham P.J. Insights from genomic profiling of transcription factors. Nat. Rev. Genet. 2009; 10:605–616. 19668247 10.1038/nrg2636 PMC2846386

[7] Vaquerizas J.M., Kummerfeld S.K., Teichmann S.A., Luscombe N.M. A census of human transcription factors: function, expression and evolution. Nat Rev. Genet. 2009; 10:252–263. 19274049 10.1038/nrg2538

[8] Lambert S.A., Jolma A., Campitelli L.F., Das P.K., Yin Y., Albu M., Chen X., Taipale J., Hughes T.R., Weirauch M.T. The human transcription factors. Cell. 2018; 172:650–665. 29425488 10.1016/j.cell.2018.01.029 PMC12908702

[9] Augello M.A., Hickey T.E., Knudsen K.E. FOXA1: master of steroid receptor function in cancer. EMBO J. 2011; 30:3885–3894. 21934649 10.1038/emboj.2011.340 PMC3209791

[10] Weedon M.N. The importance of TCF7L2. Diabet. Med. 2007; 24:1062–1066. 17888129 10.1111/j.1464-5491.2007.02258.x

[11] Tupler R., Perini G., Green M.R. Expressing the human genome. Nature. 2001; 409:832–833. 11237001 10.1038/35057011

[12] North M., Sargent C., O’Brien J., Taylor K., Wolfe J., Affara N.A., Ferguson-Smith M.A. Comparison of ZFY and ZFX gene structure and analysis of alternative 3′ untranslated regions of ZFY. Nucleic Acids Res. 1991; 19:2579–2586. 2041734 10.1093/nar/19.10.2579 PMC328173

[13] Rhie S.K., Yao L., Luo Z., Witt H., Schreiner S., Guo Y., Perez A.A., Farnham P.J. ZFX acts as a transcriptional activator in multiple types of human tumors by binding downstream of transcription start sites at the majority of CpG island promoters. Genome Res. 2018; 28:310–320. 10.1101/gr.228809.117 PMC5848610 29429977

[14] Yang H., Lu Y., Zheng Y., Yu X., Xia X., He X., Feng W., Xing L., Ling Z. shRNA-mediated silencing of ZFX attenuated the proliferation of breast cancer cells. Cancer Chemother. Pharmacol. 2014; 73:569–576. 24448637 10.1007/s00280-014-2379-y

[15] Jiang M., Xu S., Yue W., Zhao X., Zhang L., Zhang C., Wang Y. The role of ZFX in non-small cell lung cancer development. Oncol. Res. 2012; 20:171–178. 23461064 10.3727/096504012x13548165987493

[16] Jiang R., Wang J.C., Sun M., Zhang X.Y., Wu H. Zinc finger X-chromosomal protein (ZFX) promotes solid agar colony growth of osteosarcoma cells. Oncol. Res. 2012; 20:565–570. 24139414 10.3727/096504013X13775486749290

[17] Zhou Y., Su Z., Huang Y., Sun T., Chen S., Wu T., Chen G., Xie X., Li B., Du Z. The Zfx gene is expressed in human gliomas and is important in the proliferation and apoptosis of the human malignant glioma cell line U251. J. Exp. Clin. Cancer Res. 2011; 30:114. 22185393 10.1186/1756-9966-30-114 PMC3259083

[18] Fang Q., Fu W.H., Yang J., Li X., Zhou Z.S., Chen Z.W., Pan J.H. Knockdown of ZFX suppresses renal carcinoma cell growth and induces apoptosis. Cancer Genet. 2014; 207:461–466. 25441684 10.1016/j.cancergen.2014.08.007

[19] Fang X., Huang Z., Zhou W., Wu Q., Sloan A.E., Ouyang G., McLendon R.E., Yu J.S., Rich J.N., Bao S. The zinc finger transcription factor ZFX is required for maintaining the tumorigenic potential of glioblastoma stem cells. Stem Cells. 2014; 32:2033–2047. 24831540 10.1002/stem.1730 PMC4349564

[20] Nikpour P., Emadi-Baygi M., Mohammad-Hashem F., Maracy M.R., Haghjooy-Javanmard S. Differential expression of ZFX gene in gastric cancer. J. Biosci. 2012; 37:85–90. 22357206 10.1007/s12038-011-9174-2

[21] Weng H., Wang X., Li M., Wu X., Wang Z., Wu W., Zhang Z., Zhang Y., Zhao S., Liu S. et al. Zinc finger X-chromosomal protein (ZFX) is a significant prognostic indicator and promotes cellular malignant potential in gallbladder cancer. Cancer Biol. Ther. 2015; 16:1462–1470. 26230915 10.1080/15384047.2015.1070994 PMC4846125

[22] Li K., Zhu Z.C., Liu Y.J., Liu J.W., Wang H.T., Xiong Z.Q., Shen X., Hu Z.L., Zheng J. ZFX knockdown inhibits growth and migration of non-small cell lung carcinoma cell line H1299. Int. J. Clin. Exp. Pathol. 2013; 6:2460–2467. 24228108 PMC3816815

[23] Fang J., Yu Z., Lian M., Ma H., Tai J., Zhang L., Han D. Knockdown of zinc finger protein, X-linked (ZFX) inhibits cell proliferation and induces apoptosis in human laryngeal squamous cell carcinoma. Mol. Cell. Biochem. 2012; 360:301–307. 22009483 10.1007/s11010-011-1069-x

[24] Kleine-Kohlbrecher D., Christensen J., Vandamme J., Abarrategui I., Bak M., Tommerup N., Shi X., Gozani O., Rappsilber J., Salcini A.E. et al. A functional link between the histone demethylase PHF8 and the transcription factor ZNF711 in X-linked mental retardation. Mol. Cell. 2010; 38:165–178. 20346720 10.1016/j.molcel.2010.03.002 PMC2989439

[25] Rossi M.J., Lai W.K.M., Pugh B.F. Simplified ChIP-exo assays. Nat. Commun. 2018; 9:2842. 30030442 10.1038/s41467-018-05265-7 PMC6054642

[26] Chen X., Xu H., Yuan P., Fang F., Huss M., Vega V.B., Wong E., Orlov Y.L., Zhang W., Jiang J. et al. Integration of external signaling pathways with the core transcriptional network in embryonic stem cells. Cell. 2008; 133:1106–1117. 18555785 10.1016/j.cell.2008.04.043

[27] Grants J., Flanagan E., Yee A., Romaniuk P.J. Characterization of the DNA binding activity of the ZFY zinc finger domain. Biochemistry. 2010; 49:679–686. 20028140 10.1021/bi9018626

[28] Taylor-Harris P., Swift S., Ashworth A. Zfyl encodes a nuclear sequence-specific DNA binding protein. FEBS Lett. 1995; 360:315–319. 7883055 10.1016/0014-5793(95)00141-u

[29] Weirauch M.T., Yang A., Albu M., Cote A.G., Montenegro-Montero A., Drewe P., Najafabadi H.S., Lambert S.A., Mann I., Cook K. et al. Determination and inference of eukaryotic transcription factor sequence specificity. Cell. 2014; 158:1431–1443. 25215497 10.1016/j.cell.2014.08.009 PMC4163041

[30] Jones P.A. Functions of DNA methylation: islands, start sites, gene bodies and beyond. Nat. Rev. Genet. 2012; 13:484–492. 22641018 10.1038/nrg3230

[31] Miranda T.B., Jones P.A. DNA methylation: the nuts and bolts of repression. J. Cell. Physiol. 2007; 213:384–390. 17708532 10.1002/jcp.21224

[32] Swamynathan S.K. Kruppel-like factors: three fingers in control. Hum. Genomics. 2010; 4:263–270. 20511139 10.1186/1479-7364-4-4-263 PMC2975451

[33] Iuchi S. Three classes of C2H2 zinc finger proteins. CMLS, Cell. Mol. Life Sci. 2000; 58:625–635. 10.1007/PL00000885 PMC11146492 11361095

[34] Brayer K.J., Segal D.J. Keep your fingers off my DNA: protein-protein interactions mediated by C2H2 zinc finger domains. Cell Biochem. Biophys. 2008; 50:111–131. 18253864 10.1007/s12013-008-9008-5

[35] Jen J., Wang Y.C. Zinc finger proteins in cancer progression. J. Biomed. Sci. 2016; 23:53. 27411336 10.1186/s12929-016-0269-9 PMC4944467

[36] Liang S., Jiang N., Li S., Jiang X., Yu D. A maternally inherited 8.05 Mb Xq21 deletion associated with Choroideremia, deafness, and mental retardation syndrome in a male patient. Mol. Cytogenet. 2017; 10:23. 28630650 10.1186/s13039-017-0324-6 PMC5471966

[37] van der Werf I.M., Van Dijck A., Reyniers E., Helsmoortel C., Kumar A.A., Kalscheuer V.M., de Brouwer A.P., Kleefstra T., van Bokhoven H., Mortier G. et al. Mutations in two large pedigrees highlight the role of ZNF711 in X-linked intellectual disability. Gene. 2017; 605:92–98. 27993705 10.1016/j.gene.2016.12.013

[38] Jin Z., Yu L., Geng J., Wang J., Jin X., Huang H. A novel 47.2 Mb duplication on chromosomal bands Xq21.1-25 associated with mental retardation. Gene. 2015; 567:98–102. 25956375 10.1016/j.gene.2015.04.083

[39] Levens D. Disentangling the MYC web. Proc. Natl. Acad. Sci. U.S.A. 2002; 99:5757–5759. 11983876 10.1073/pnas.102173199 PMC122847

[40] Holzel M., Kohlhuber F., Schlosser I., Holzel D., Luscher B., Eick D. Myc/Max/Mad regulate the frequency but not the duration of productive cell cycles. EMBO Rep. 2001; 2:1125–1132. 11743027 10.1093/embo-reports/kve251 PMC1084169

[41] Hall T.M. Multiple modes of RNA recognition by zinc finger proteins. Curr. Opin. Struct. Biol. 2005; 15:367–373. 15963892 10.1016/j.sbi.2005.04.004

[42] Theunissen O., Rudt F., Guddat U., Mentzel H., Pieler T. RNA and DNA binding zinc fingers in Xenopus TFIIIA. Cell. 1992; 71:679–690. 1423623 10.1016/0092-8674(92)90601-8

[43] Brannan K.W., Jin W., Huelga S.C., Banks C.A., Gilmore J.M., Florens L., Washburn M.P., Van Nostrand E.L., Pratt G.A., Schwinn M.K. et al. SONAR discovers RNA-binding proteins from analysis of large-scale protein-protein interactomes. Mol. Cell. 2016; 64:282–293. 27720645 10.1016/j.molcel.2016.09.003 PMC5074894

[44] Saldana-Meyer R., Rodriguez-Hernaez J., Escobar T., Nishana M., Jacome-Lopez K., Nora E.P., Bruneau B.G., Tsirigos A., Furlan-Magaril M., Skok J. et al. RNA interactions are essential for CTCF-mediated genome organization. Mol. Cell. 2019; 76:412–422. 31522988 10.1016/j.molcel.2019.08.015 PMC7195841

[45] Hansen A.S., Hsieh T.S., Cattoglio C., Pustova I., Saldana-Meyer R., Reinberg D., Darzacq X., Tjian R. Distinct classes of chromatin loops revealed by deletion of an RNA-binding region in CTCF. Mol. Cell. 2019; 76:395–411. 31522987 10.1016/j.molcel.2019.07.039 PMC7251926

[46] Moran S., Arribas C., Esteller M. Validation of a DNA methylation microarray for 850,000 CpG sites of the human genome enriched in enhancer sequences. Epigenomics. 2016; 8:389–399. 26673039 10.2217/epi.15.114 PMC4864062

