# Supplementary material for: Characterization of the ZFX family of transcription factors that bind downstream of the start site of CpG island promoters
Source: Nucleic Acids Res. 2020 May 14;48(11):5986–6000. doi: 10.1093/nar/gkaa384 (PMC7293018; doi:10.1093/nar/gkaa384)
Supplement: gkaa384_Supplemental_Files [file gkaa384_supplemental_files.zip › Ni.SupplementaryFigures.pdf]

Ni. Figure S1

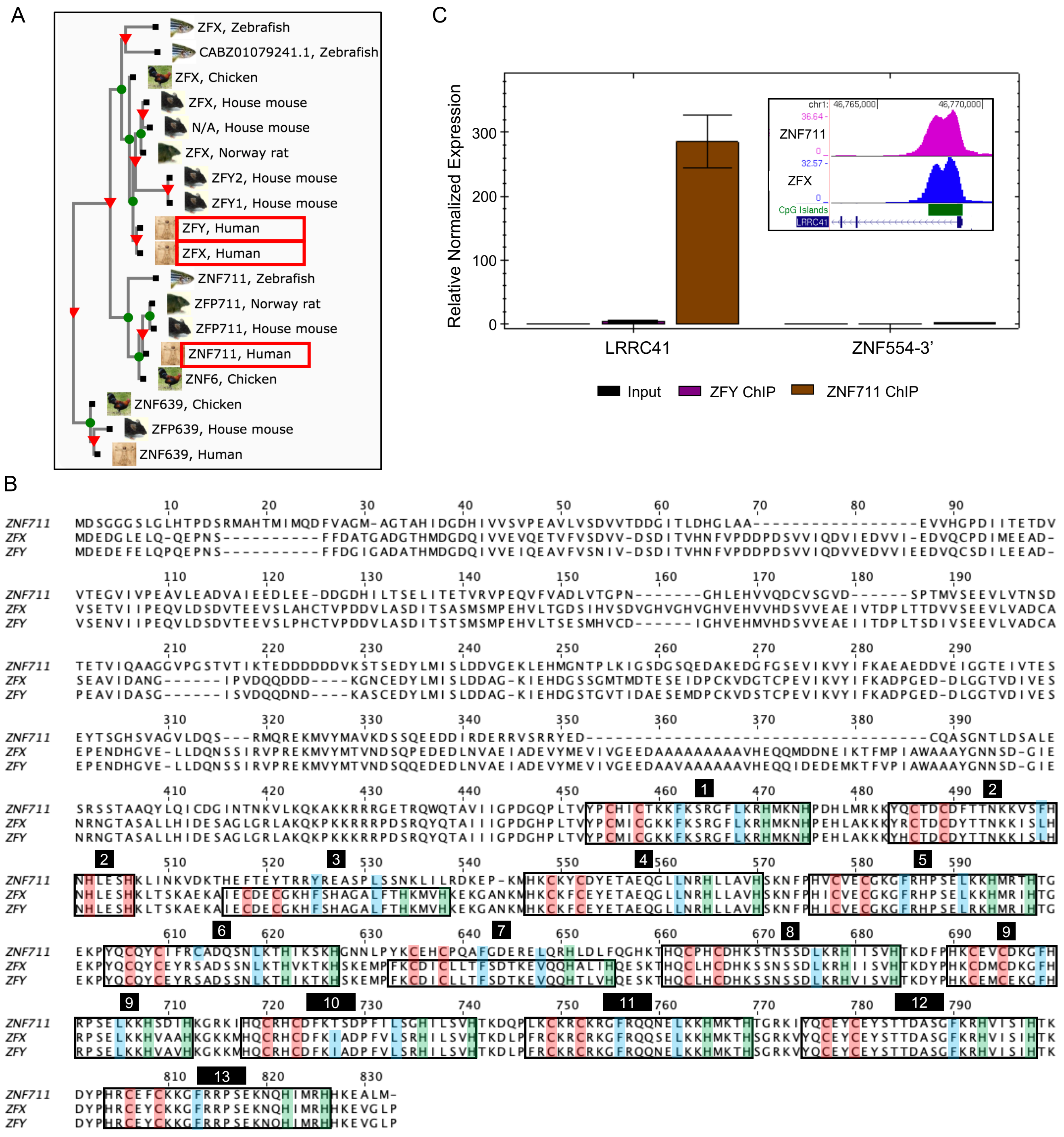

Figure S1. Amino acid alignment of the ZFX family members. A) Shown is a Treefam (www.treefam.org/) alignment for ZFX, identifying the closely related ZFY and ZNF711 proteins. **B)** Alignments were generated using sequences (ZFX-P17010, ZFY-P08048, ZNF711-Q9Y462) from UniProt (www.uniprot.org), aligned using MUSCLE (www.ebi.ac.uk/Tools/msa/muscle/), output was generated in ClustalW format (www.genome.jp/tools-bin/clustalw), and viewed using Jalview v2.10.4 (jalview.org). Each of the 13 C_2H_2 zinc fingers of ZFX are boxed; for each finger, the cysteines are marked in red and the histidines are marked in green; cysteines and histidines not related to the zinc finger structure are not color coded. Optimal DNA binding zinc fingers have the structure X-C-X_{2-5}-C-X_3-ψ-X_5-ψ-X_2-H-X_{3-5}-H (where ψ is a hydrophobic amino acid); if present in the right position, each ψ is marked in blue. **C)** ChIP assays were performed using an antibody to ZFY or ZNF711 in female HEK293T cells and binding was analyzed using primers for a known ZFY and ZNF711 binding site (LRRC41) and a negative region (ZNF554-3'). Although ZNF711 showed very robust binding to LRRC41, ZFY binding was not detected. For comparison, the inset shows that ZFX binding to the LRRC41 promoter is detected at similar levels as ZNF711 binding.

Ni. Figure S2

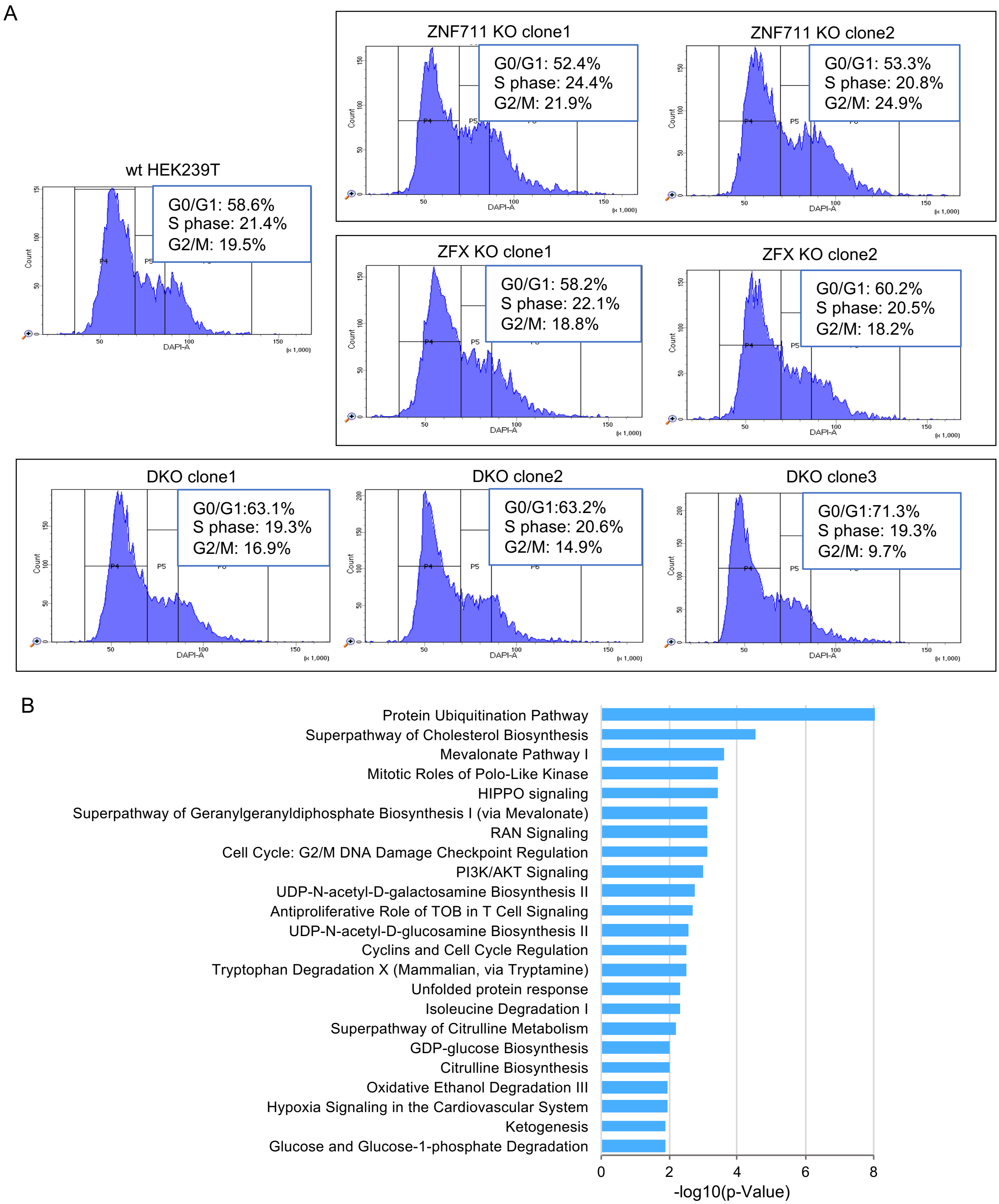

Figure S2. Cell cycle analysis of knockout cells and pathway analysis of downregulated genes in all 3 DKO clones A) Cells (wt HEK293T, two ZFX KO clones, two ZNF711 KO clones, and three DKO clones) were fixed, labelled with DAPI (10 ug/mL), and analyzed using BD LSR II gating on single cells via Width and Area signals. The percentages of G0/G1, S, and G2/M phases were calculated from the DAPI-area histogram. **B)** The top identified pathways for the set of genes commonly downregulated in all 3 DKO clones is shown.

Ni. Figure S3

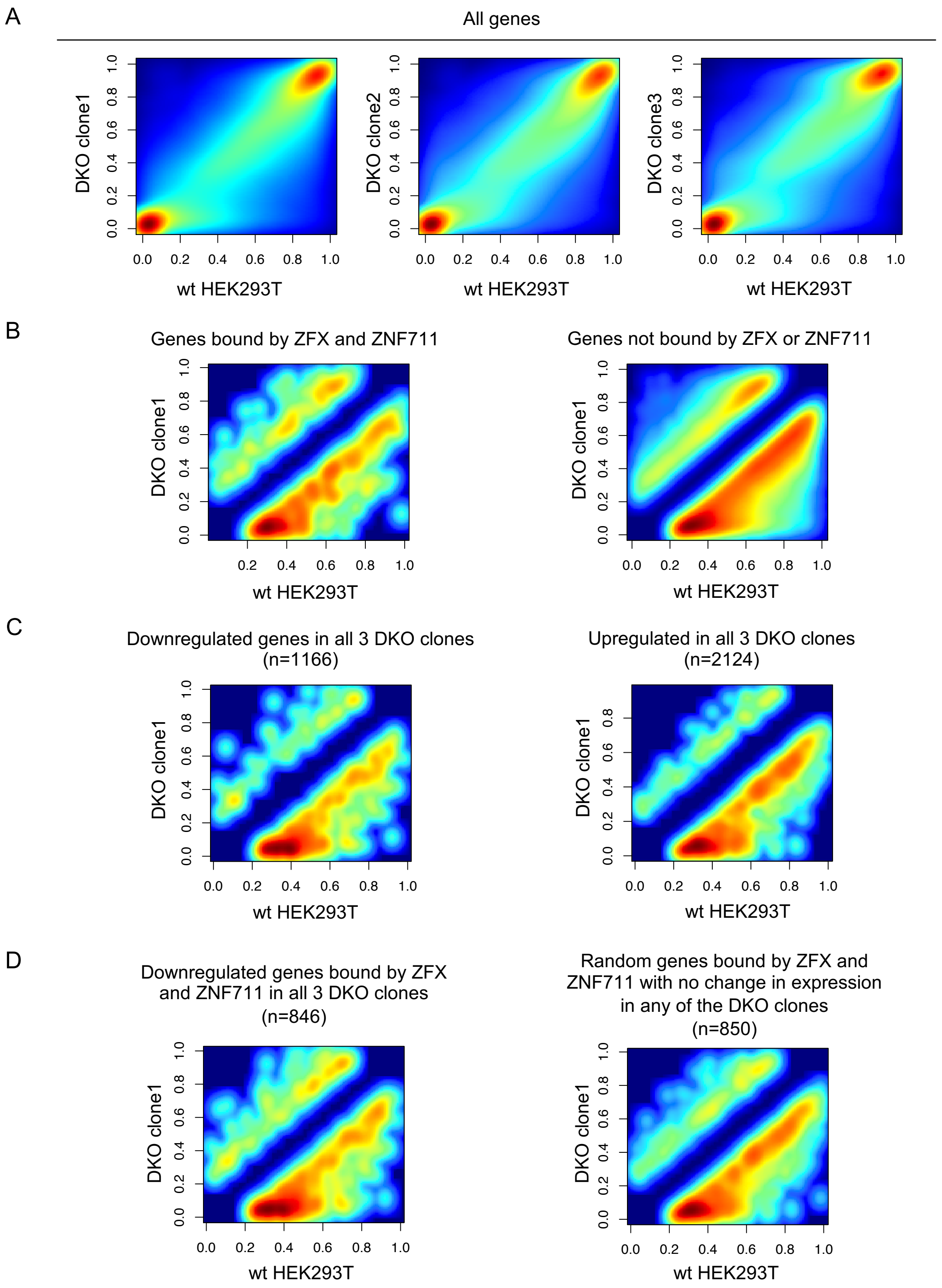

Figure S3. DNA methylation analysis of DKO cells. A) Shown is the DNA methylation level at all promoter probes on the EPIC array for each of the 3 DKO clones. To more clearly visualize any hypermethylated or hypomethylated promoter probes, probes with beta value differences in DKO vs. wt HEK293T < 0.2 were removed for panels B, C, and D. **B)** Shown is the DNA methylation level at all promoters on the array that are bound vs. not bound by ZFX and ZNF711 in wt HEK293T. **C)** Shown is the DNA methylation level of promoters that are downregulated or upregulated in DKO cells (binding by ZFX and ZNF711 was not taken into consideration). **D)** Shown is the DNA methylation level at promoters of genes that are bound by ZFX and ZNF711 and downregulated in all 3 DKO clones vs. bound by ZFX and ZNF711 but show no change in expression in DKO cells. Note that Panel B, C, and D show results of using data from DKO clone1, but very similar patterns were seen for all 3 DKO clones. In summary, all 3 of the DKO clones show a modest hypomethylation at many promoters, independent of whether the promoters are direct target of ZFX or ZNF711.

Ni. Figure S4

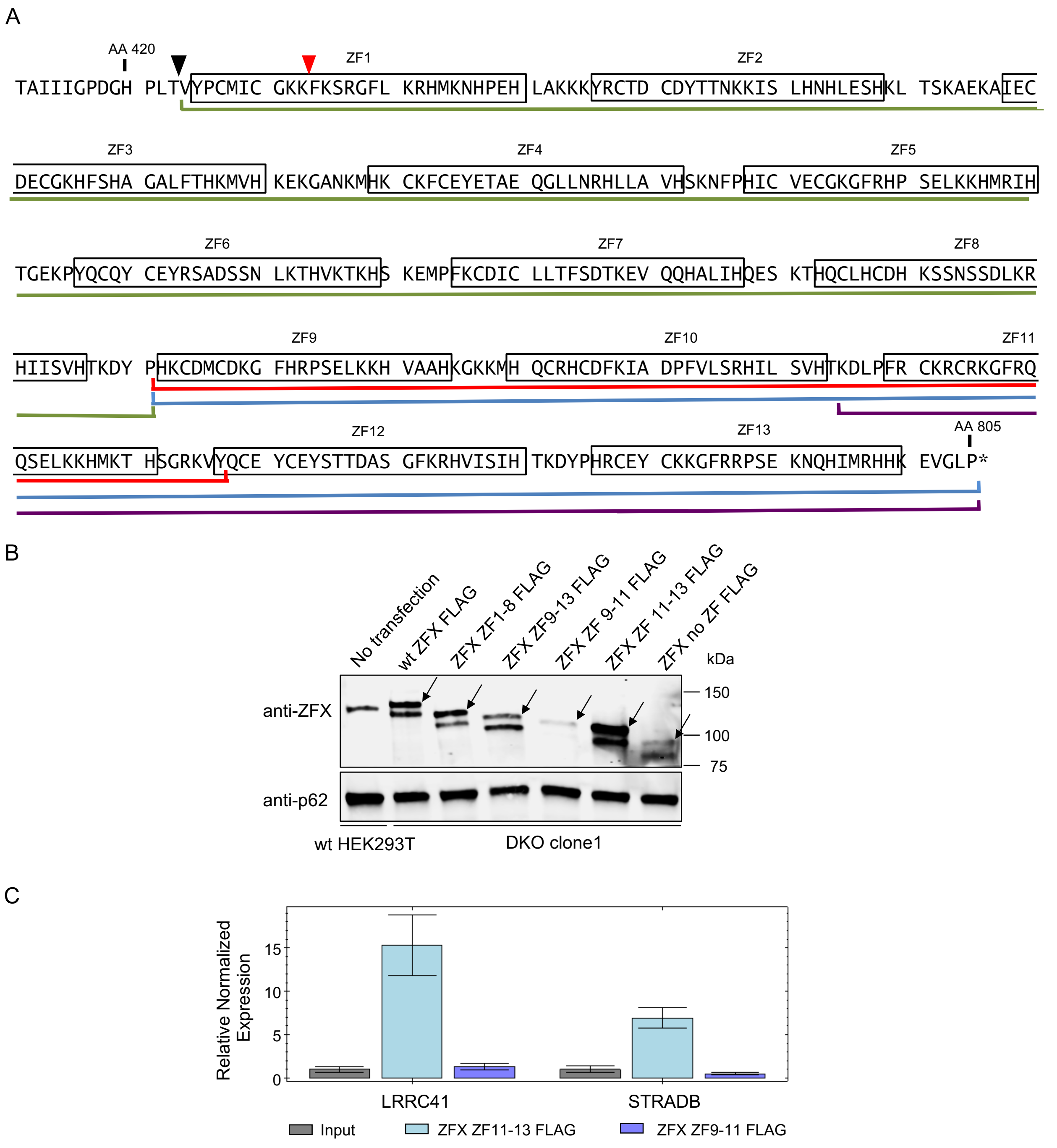

Figure S4. Sequences and protein levels of ZFX zinc finger (ZF) mutant constructs. A) Peptide sequences used in the zinc finger mutants. Only the zinc finger-containing portion of ZFX from amino acids 411 to 805 (UniProtKB- P17010 designation) is shown for clarity. The positions of the 13 ZFs are indicated by numbered boxes. Colored underlines indicate the extent of the regions present in the expression constructs: green, ZF1-8; blue, ZF9-13; red, ZF9-11; purple, ZF11-13. The black triangle indicates the position of the last ZFX amino acid present in the no zinc finger mutant and the red triangle shows the junction point between the N-terminal half of the protein and the ZF regions in mutants ZF9-13, ZF9-11, and ZF11-13. **B)** Shown is an anti-ZFX Western blot demonstrating the protein expression levels of endogenous ZFX in wt HEK293T cells and ZFX ZF mutant constructs in DKO cells; p62 was used as a loading control. The doublets in the FLAG ZFX lanes could be due to conformational differences, protein modifications, or degradation products. We note that in this particular transfection, the ZFX ZF9-11 is expressed at lower levels than the other mutants; however, in other transfections it was expressed as high as the full length ZFX. **C)** ChIP-qPCR analysis of ZF mutant enrichment at promoter regions. wt HEK293T cells were transfected with FLAG-tagged ZFX mutant constructs and ChIPs were performed using a FLAG antibody. Shown are qPCR results using ChIP DNA demonstrating that ZFX ZF11-13 FLAG can bind to target promoters. LRRC41 and STRADB promoters are bound by endogenous ZFX in wt HEK293T cells; ZNF180 was used a negative control.

Ni. Figure S5

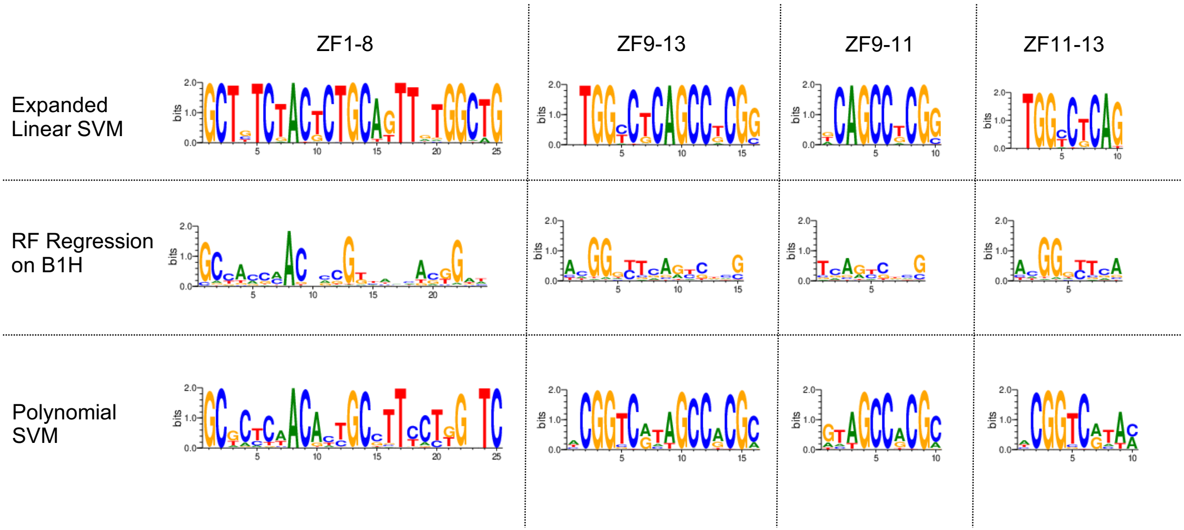

Figure S5. Motif predictions for ZFX zinc fingers. Different subsets of ZFX zinc fingers corresponding to the mutants we generated were analyzed using the website tool called DNA-binding Specificities of Cys_2His_2 Zinc Finger Proteins (http://zf.princeton.edu/); see also NAR, 42(3): 1497-1508; NAR, 43. Epub 2015 Jan 15. Each subset of zinc fingers was analyzed using 3 different methods. The ZFX protein sequence used was P17010 (www.uniprot.org/). For comparison, the motif identified using ZFX and ZNF711 ChIP-exo datasets is GGCCT.

Ni. Fig S6

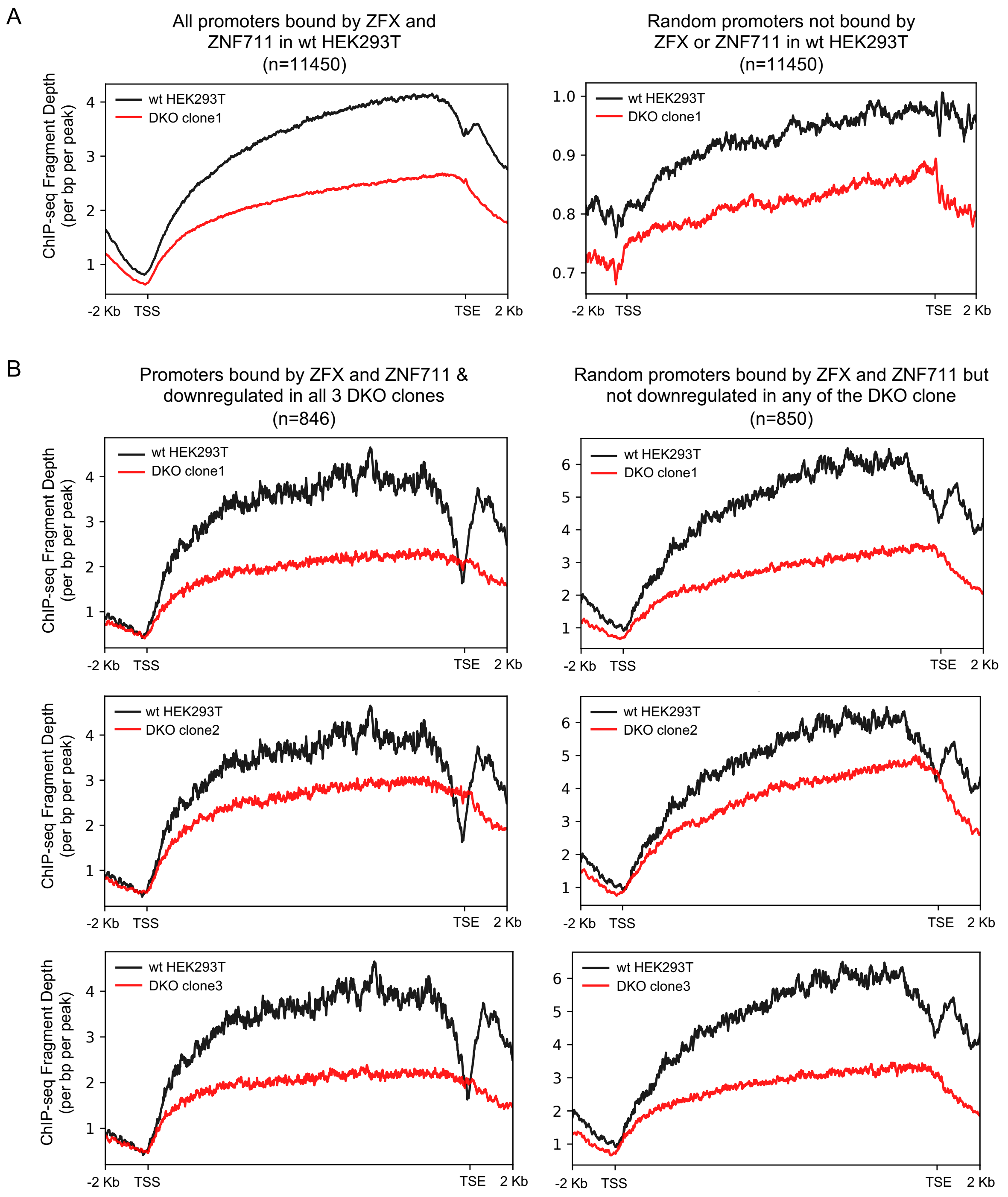

Figure S6. H3K36me3 analyses in wt HEK293T and DKO clones A) Metagene plots showing the tag density of H3K36me3 across genes that are bound by ZFX and ZNF711 in wt HEK293T (left) and random genes that are not bound by either ZFX or ZNF711 (right) in wt HEK293T and DKO clone1. **B)** Metagene plots in wt HEK293T and DKO clone1, DKO clone2, or DKO clone3 showing the tag density of H3K36me3 across genes whose promoters are bound by ZFX and ZNF711 & down-regulated in all 3 DKO clones (left) and random genes whose promoters are bound by ZFX and ZNF711 but not downregulated in any of the DKO clones (right).
